# Privacy and Smart Speakers in Research With Older Adults

**DOI:** 10.1093/geroni/igab046.994

**Published:** 2021-12-17

**Authors:** Kelly Quinn, Jessie Chin, Smit Desai, Carrie O'Connell, David Marquez, Naoko Muramatsu

**Affiliations:** 1 University of Illinois at Chicago, Chicago, Illinois, United States; 2 University of Illinois at Urbana-Champaign, University of Illinois at Urbana-Champaign, Illinois, United States; 3 University of Illinois, Urbana Champaign, Champaign, Illinois, United States; 4 University of Illinois at Chicago, University of Illinois at Chicago, Illinois, United States; 5 University of Illinois at Chicago & Rush University, University of Illinois at Chicago, Illinois, United States; 6 University of Illinois Chicago, Chicago, Illinois, United States

## Abstract

Advances in artificial intelligence and computational linguistics have made smart speakers, such as Amazon Alexa^TM^ and Google Home^TM^, economical and widely available. For older adults particularly, devices with voice interfaces can help to overcome accessibility challenges that often accompany interaction with today’s technologies. However, voice-activation also requires devices to be in a continuous state of ambient listening, which can create a significant privacy risk for the user, one that is often amplified as smart speakers are placed in highly personal home spaces to facilitate their utility. Deployment of these devices in research settings poses additional risk, as traces of data filter through research teams, app developers, and third-party services that support research efforts. This presentation addresses the privacy aspects of deploying Google Home Mini^TM^ speakers in research that examined their feasibility for enhancing physical activity among sedentary older adults. Interviews with participants were conducted in two studies: the first included a demonstration of the device and physical activity program (n=15); and the second included in-home use of devices and a physical activity program (n=15). Content analysis of study documentation, field notes, and interviews revealed specific areas that require additional attention when utilizing smart speakers in research, including the capture of identifying information, protocols for data handling, and requirements for informed consent. These findings are discussed in context with extant literature on individual privacy concerns and behaviors related to smart household devices. Results from this study can inform future research efforts incorporating smart speakers, to mitigate potential risks of privacy violation.

